# 周围型肺癌干性胸膜转移的多层螺旋CT影像学诊断

**DOI:** 10.3779/j.issn.1009-3419.2014.05.07

**Published:** 2014-05-20

**Authors:** 佳 刘, 文武 李, 勇 黄, 聿辉 刘

**Affiliations:** 1 250017 济南，山东省医学科学院，山东省肿瘤防治研究院放射科 Department of Radiology, Shandong Tumor Hospital, Affilialed to Shandong Acadaemy of Medical Sciences, Jinan 250017, China; 2 250007 济南，济南大学，山东省医科院医学与生命科学学院 School of Medicine and Life Scineces, University of Jinan-Shandong Acadamy of Medical Scineces, Jinan 250007, China

**Keywords:** 肺肿瘤, 干性胸膜转移, X线计算机体层摄影术, Lung neoplasms, Dry pleural dissemination, X-ray computed tomography

## Abstract

**背景与目的:**

周围型肺癌的胸膜转移率较高，且干性胸膜转移术前容易漏诊，造成不必要的手术，因此术前诊断就尤为重要。回顾性分析伴有干性胸膜转移的周围型肺癌的多层螺旋CT（multislice spiral computed tomography, MSCT）影像，并探讨其对干性胸膜转移的诊断价值。

**方法:**

对27例经病理或临床证实的周围型肺癌伴有干性胸膜转移的MSCT影像学表现进行回顾性分析。

**结果:**

本组27例干性胸膜转移，CT检出率为85%，叶间胸膜转移检出率为91%，非叶间胸膜检出率为63%。27例中多发胸膜结节者26例（96%），所有胸膜结节均位于病变同侧，有叶间胸膜结节者23例，多表现为小结节，可沿叶间胸膜呈串珠样或簇状排列；有非叶间胸膜结节者8例，以大结节多见，肺-结节界面清晰；27例中胸膜增厚者15例（56%），可表现为胸膜带状增厚、不均匀增厚或两者同时存在。本组干性胸膜转移者影像学表现以混合型多见（63%）。

**结论:**

MSCT对周围型肺癌患者干性胸膜转移有较好的诊断价值，尤其对表现为胸膜结节者有较高的准确性。

## Abstract (en)

**Background and objective:**

The rate of pleural metastasis in peripheral lung cancer is high, and the dry pleural metastasis easily missed diagnosis preoperatively and cause unnecessary surgery. Therefore, preoperative diagnosis is particularly important. To review the multislice spiral computed tomography (MSCT) image of peripheral lung cancer with dry pleural dissemination, and to discuss its diagnostic value for understanding the dry pleural dissemination.

**Methods:**

Reviewed and analyzed the MSCT images of pathologically or clinically diagnosed peripheral lung cancer with dry pleural dissemination in 27 patients. Analyze the imaging characteristics respectively from pleural thickening and pleural nodules.

**Results:**

The dry pleural dissemination of lung cancer were detected in 85% by CT. The rate of CT detection of dissemination on the visceral pleura and the interlobar pleura were 63% and 91%, respectively. 26 cases were with multiple pleural nodules, all were located on the same side with the primary lesions; 8 cases were with peritoneum visceralis nodules that are mostly circular with diameters of 3 mm-15 mm. The lung-nodules interfaces were clear. 23 cases had interlobar pleura nodules (all with more than 6 nodules), some are big (diameter > 5 mm) while some are small (diameter < 5 mm). The nodules are arranged along the interlobar pleura as beaded string or in clusters around the interlobar pleura. 15 cases were with pleural thickening, including band-like, uneven, or both exist at the same time. There are more mixed type in this group (63%).

**Conclusion:**

MSCT has great diagnostic value for peripheral lung cancer with dry pleural dissemination, especially with high accuracy of pleural nodules.

肺癌的胸膜转移分为湿性胸膜转移（wet pleural dissemination, WPD）和干性胸膜转移（dry pleural dissemination, DPD）。恶性胸腔积液提示存在湿性胸膜转移，而干性胸膜转移是指非小细胞肺癌无胸腔积液的胸膜转移^[1]^。后者属于肺癌Ⅳ期，应以全身治疗为主要治疗手段^[2]^，但因其不伴有胸腔积液，所以术前可能漏诊而导致不必要的手术^[3]^。文献报道螺旋CT（computed tomography）检查有助于检出肺癌患者的胸膜转移，薄层螺旋CT扫描对其诊断的准确性可达90%^[2]^。现国内对干性胸膜转移的CT影像学表现、尤其多层螺旋CT（multislice spiral CT, MSCT）未见报道。因此笔者收集27例经病理证实或临床诊断的伴有干性胸膜转移的肺癌患者，对其MSCT的影像学表现进行回顾性分析，探讨其CT影像学特征，以期提高对干性胸膜转移的检出率及诊断水平。

## 1 材料与方法

### 1.1 病例资料

收集本院自2009年10月-2012年12月经病理证实或临床诊断为有胸膜转移无显性胸水的非小细胞肺癌患者27例。所有患者均经病理证实为非小细胞肺癌（其中手术者8例，纤维支气管镜者4例，CT引导下穿刺者15例）。本组行手术治疗或胸膜病灶穿刺者经病理证实为胸膜转移；未行手术治疗或穿刺者，结合肺癌病史，其胸膜病灶进行性增大或胸膜病灶抗肿瘤治疗后好转者则临床诊断为胸膜转移。年龄36岁-75岁，平均年龄（57.8±6.72）岁。男16例，女11例。原发灶长径20 mm以下4例，20 mm-30 mm 18例，30 mm以上5例。所有患者均行MSCT增强扫描。回顾性分析患者术前MSCT影像学表现及检查报告，其中术前未检出有胸膜转移的有4例，回顾性分析其薄层重组图像，有2例在薄层CT图像中可见叶间胸膜结节。

### 1.2 影像学检查方法

对27例患者行MSCT平扫和增强扫描，CT机采用PHILIPS ICT机行螺旋扫描。扫描条件120 kV，200 mAs，原始采集层厚0.625 mm，重建层厚5 mm，螺距1.5。增强扫描：静脉团注非离子型对比剂碘葡胺80 mL-100 mL，剂量1.5 mL/kg，注射速度2.5 mL/s，经肘静脉高压注射。扫描时患者空腹平卧，屏气扫描，扫描范围自肺尖至膈顶。常规厚层图像传至工作站进行图像后处理：横断面薄层重建，重建参数：PHILIPS ICT，层厚1.5 mm，间隔0.75 mm或2 mm层厚，间隔1 mm。部分病例行冠状位及矢状位多平面图像重组（multi-planar reformation, MPR）。

### 1.3 观察内容

由两名高年资主治及副主任医师对其影像学表现进行回顾性分析。分别依叶间胸膜和非叶间胸膜（肋胸膜、纵隔胸膜、膈胸膜）进行观察。观察内容包括：①有无显性胸水，②有无胸膜结节，③有无胸膜增厚，并记录胸膜结节的位置、大小、个数以及胸膜增厚情况，集中讨论归纳其影像学表现特点。并根据其影像学表现进行分型，分为：①大结节型：脏层胸膜结节（长径） > 5 mm者，②小结节型：脏层胸膜结节（长径） < 5 mm者，③条带型：脏层胸膜带状或不均匀增厚，④混合型：具备上述两种或以上类型。

## 2 结果

27例肺癌患者（全部为周围型肺癌），经病理证实或临床诊断为胸膜转移（经手术或穿刺病理证实；无病理者其胸膜病灶进行性增大或胸膜病灶抗肿瘤治疗后好转者则临床诊断为胸膜转移），CT诊断为胸膜转移者23例（85%）。其中叶间胸膜转移23例，检出21例（91%），非叶间胸膜转移11例，检出7例（63%），

### 2.1 原发灶的特征

#### 2.1.1 原发灶与胸膜的关系

原发灶与胸膜关系相贴者17例（63%），有胸膜牵拉征者9例（33%），与胸膜无关者2例（4%）。

#### 2.1.2 原发灶的病理类型

原发灶的病理经纤维支气管镜、CT引导穿刺或手术证实。27例中，22例有原发灶的具体病理学类型，腺癌20例（91%），腺鳞癌2例（9%），其余5例病理诊断为非小细胞癌，具体分类不明。

### 2.2 干性胸膜转移的影像学表现

所有胸膜病灶均位于病变同侧。27例中，出现脏层胸膜结节者26例。其中非叶间胸膜结节者8例（肋胸膜6例，纵隔胸膜1例，膈胸膜1例），以大结节（长径 > 5 mm）多见，大多表现为类圆形，大小（长径）为3 mm-15 mm，肺-结节界面清晰。叶间胸膜结节者23例，以小结节（长径 < 5 mm）多见，可沿叶间胸膜呈串珠样排列或在叶间胸膜周围呈簇状分布（图 1）。所有病例叶间胸膜结节数量最少者为7个，胸膜结节数目：1个-6个者0例，6个-10个者4例，10个-20个者8例，20个以上者11例。

**1:**
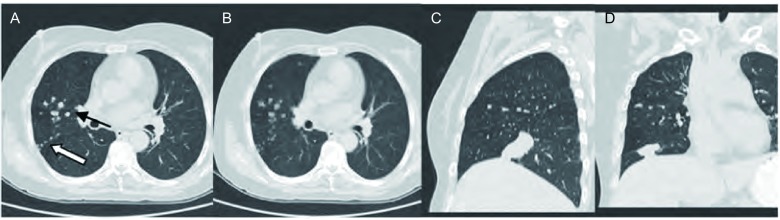
患者女，60岁，右肺下叶腺癌伴干性胸膜转移（叶间胸膜小结节型）。A、B：右侧叶间胸膜多发小结节，结节部分沿右肺水平裂呈簇状分布，部分沿右肺斜裂排列呈串珠状排列（层厚分别为5 mm和2 mm）。C、D（同一患者MPR）：原发灶位于右肺下叶，并与右肺斜裂关系相贴；右侧叶间胸膜小结节，沿右肺水平裂呈串珠状排列（层厚分别为5 mm和2 mm）。 Female, 60-year-old computed tomography (CT) images show dry pleural dissemination (DPD) (the small interlobar pleura nodules) in 60-year-old woman with lung adenocacinoma. A, B: Lung window of CT image (5-mm, 2-mm section thickness) shows multiple small interlobar pleura nodules within right minor and major fissures which arranged along the interlobar pleura as beaded string or in clusters around the interlobar pleura. C, D: The primary tumor in the right lower lobe is visualized, and located adjacent to the right major fissure. CT images (5-mm, 2-mm section thickness) show multiple small interlobar pleura nodules which arranged along the right minor fissure as beaded string.

27例中，出现胸膜增厚者15例（56%），其中带状增厚者5例（叶间胸膜4例，非叶间胸膜1例），不均匀增厚者11例（叶间胸膜2例，非叶间胸膜9例），部分病例两者同时出现。胸膜增厚常伴胸膜结节，单纯表现为胸膜增厚者仅1例。

27例胸膜转移者中：①大结节型2例（图 2），占8%（肋胸膜结节1例，叶间胸膜结节1例）；②小结节型7例，占25%（全部为叶间胸膜结节）；③条带型1例，占4%（肋胸膜不均匀增厚）；④混合型17例，占63%（①+③：2例，结节全部表现为非叶间胸膜结节；②+③：6例，膈胸膜结节1例，叶间胸膜结节5例；①+②：3例，结节全部表现为叶间胸膜结节；①+②+③：6例）。

**2:**
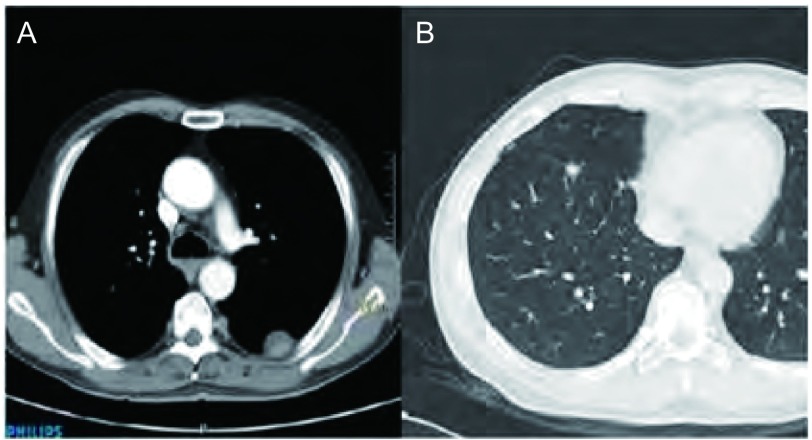
大结节型。A：患者男，59岁，CT引导下穿刺病理示低分化腺癌。左侧肋胸膜结节，长径约25 mm的类圆形大结节，肺-结节界面清晰；B：患者女，66岁，右肺腺癌；右侧叶间胸膜结节，长径约9 mm的圆形孤立性大结节。 Type of large nodule. A: CT images show a quasi-circular nodule within the right costal pleura in 59-year-old man with lung poorly differentiated adenocarcinoma confirmed by CT guided biopsy pathology, diameters about 25-mm and the lung-nodules interfaces were clear; B: CT images show a circular solitary nodule within the right major fissure in 66-year-old woman with lung adenocarcinoma, diameters about 9-mm.

### 2.3 随诊

有12例患者随诊6个月-14个月。其中胸膜结节结节未变化者（随诊6个月）1例；结节进行性增大者8例；1例由结节型改变为条带型；2例随诊中出现恶性胸腔积液（图 3）。

**3:**
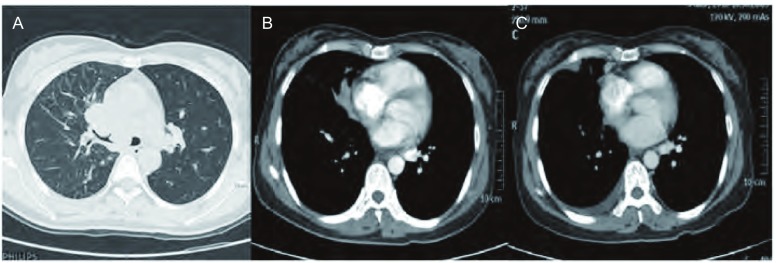
患者女，43岁，肺腺癌伴干性胸膜转移进展为湿性胸膜转移。A：原发灶位于右肺上叶，伴同侧斜裂多发结节状转移；B：胸膜腔内未见积液；C：6个月后，右侧胸腔内示弧形液性密度影，胸水中查到腺癌细胞。 Femal, 43-year-old, follow-up CT images show DPD with eventual pleural effusion in 43-year-old woman with lung adenocarcinoma. A: The primary tumor located in the right upper lobe, with multiple nodal metastases within the ipsilateral major fissure; B: Mediastinal window of CT image (5-mm section thickness) shows no pleural effusion; C: CT image obtained at a similar level and 6 months after A shows a small amount of right pleural effusion, and check lung adenocarcinoma cell by hydrothorax exfoliative cytologic examination.

## 3 讨论

肺癌侵袭性较强，非小细胞肺癌胸膜转移的发生率较高，有研究^[4]^表明临近胸膜的肺癌其胸膜转移发生率高达37%。第七版非小细胞肺癌的TNM分期已将胸膜转移由T4移至M1a，包括胸膜结节和恶性胸膜播散^[2]^。M1a期的肺癌患者应采取以放化疗为主的全身治疗。而MSCT扫描可对肺癌患者的胸膜转移做出及时、准确的诊断，从而避免不必要的手术治疗。干性胸膜转移因其无显性胸水，更易造成漏诊，术前及时检出就显得尤为重要。近年来16层以上的MSCT已成为胸部影像学检查的主流，其空间分辨率明显高于传统CT，使微小的胸膜转移灶的检出成为可能。

### 3.1 原发灶的特征与干性胸膜转移的关系

在本组有明确病理学分型的22例肺癌中，病理类型为腺癌或腺鳞癌，提示干性胸膜转移主要见于腺癌。Kim^[5]^的研究中也显示，在其研究的存在胸膜转移的原发性肺癌患者中，约90%的患者病理类型为腺癌。本组27例中，原发灶与胸膜相贴者17例（63%），有胸膜牵拉征者9例（33%），与胸膜无关者仅2例（4%）。与之前文献报道相符，Murayama等^[6]^指出干性胸膜转移好发于原发病灶与胸膜或叶间胸膜相贴的肺癌。因此，在初诊患者病理及细胞学证实原发灶为腺癌且病变与胸膜关系密切者，必须仔细观察胸膜是否存在转移以避免漏诊，以免对临床治疗产生误导。本组未发现初诊为鳞癌或其他病理类型的非小细胞癌出现胸膜转移，但这需要更大宗病例的证实。反之，对于出现干性胸膜转移的肺癌，即使在取得病理学结果之前，也高度提示其病理类型为腺癌。

### 3.2 干性胸膜转移的影像学表现

据以往文献^[6-8]^报道，干性胸膜转移的主要影像学表现包括：叶间胸膜或其他脏层胸膜结节及带状或不均匀的胸膜增厚。肺内淋巴结、矽肺结节、肉芽肿及肺纤维灶与干性胸膜转移影像学表现相似，详细的病史及接触史有助于鉴别^[1, 9]^。本组大多数病例（23/27; 85%），MSCT都检出了干性胸膜转移。未能检出的病例可能与胸膜转移灶较小有关。

本组中所有的干性胸膜转移均位于病变同侧，对侧均未见胸膜转移，提示干性胸膜转移常见于同侧。因此，对于肺癌患者，我们应着重于观察同侧的脏层胸膜，而仅在原发灶对侧出现的胸膜结节很可能为良性结节而不是转移。27例干性胸膜转移中，23例（85%）存在叶间胸膜转移，无叶间胸膜转移者仅4例，提示叶间胸膜为干性胸膜转移的高发区域，必须引起高度警惕。

干性胸膜转移的影像学表现为胸膜结节及胸膜增厚，以前者较为常见，而胸膜结节又以叶间胸膜结节多见，这可能为叶间胸膜结节因含气肺组织的衬托更易被观察到有关。叶间胸膜结节通常仅能在肺窗发现，但当其呈大结节型，部分可在纵隔窗显示，增强扫描可见强化。干性胸膜转移的胸膜结节影像学表现为：①非叶间胸膜结节，大小不一，以大结节（长径 > 5 mm）多见。②叶间胸膜结节（结节数量均 > 6个^[1]^）：大结节（长径 > 5 mm）者较少见，可表现为孤立性或沿叶间胸膜排列，部分周围可见数个小结节；小结节（长径 < 5 mm）者较常见，可表现为沿叶间胸膜呈串珠样排列或在叶间胸膜周围呈簇状分布。

有研究^[1]^证实，当患者CT扫描出现 > 6个的叶间胸膜或其他脏层胸膜结节（100%）或不均匀胸膜增厚（50%），其可被认为是非小细胞肺癌患者存在干性胸膜转移的特征性影像学表现。因此当原发灶与脏层胸膜关系密切时，应着重观察其有无胸膜结节或胸膜增厚，尤其要重点观察同侧，必要时可行薄层重建肺窗，当其出现多发胸膜结节且数目 > 6个时^[1]^，高度提示存在干性胸膜转移。数目 < 6个者，良性结节的可能性较大。对于薄层重建我们应引起足够的重视，有些叶间胸膜结节在常规层厚图像（5 mm）显示不清，仅在薄层图像中显示，本组中有2例叶间胸膜转移结节（微小胸膜转移）仅在薄层图像中显示（图 4）。薄层扫描（层厚一般为1 mm-2 mm）和高分辨率的图像重组算法有助于提高非小细胞肺癌患者干性胸膜转移诊断的敏感度和特异度。因此，薄层扫描CT应作为非小细胞肺癌患者干性胸膜转移的首选检查方法^[7]^。

**4:**
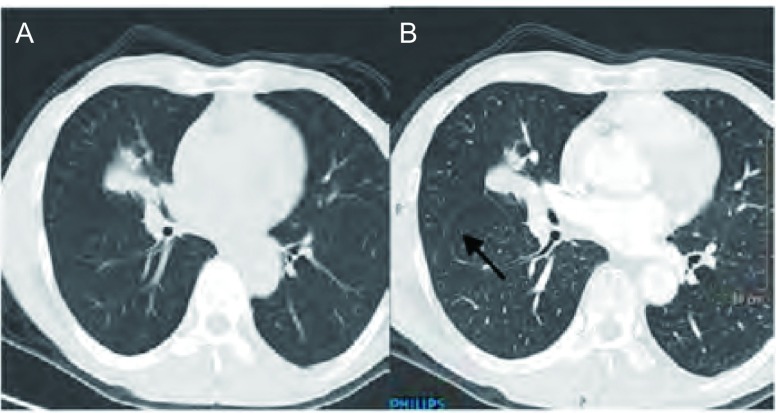
原发灶位于右肺，手术病理证实原发灶为中分化腺癌。A、B：手术病理证实右肺水平裂微小叶间胸膜转移，同一层面，层厚分别为5 mm、1.5 mm。5 mm层厚未见胸膜结节，1.5 mm层厚清晰显示胸膜结节（箭头）。 The primary tumor located in the right lower lobe, and were confirmed to be moderately differentiated adenocarcinoma pathologically by surgical. The micrometastasis within the right minor fissure were confirmed pathologically. A: Lung window of CT image (5-mm section thickness) shows no nodules; B: CT images (1.5-mm section thickness) obtained at the same levels shows multiple small right minor fissural nodules.

本组干性胸膜转移者以混合型多见（17/27, 63%），胸膜结节与胸膜增厚常伴发，仅表现为胸膜增厚者更为少见。大结节型多表现为胸壁胸膜结节，可能与小的胸壁胸膜结节不易观察有关。而小结节型多表现为叶间胸膜结节，尤其是肺窗中更易观察到，这可能与其较高的对比度有关。条带型少见（1/27, 4%）。通过随访部分病例，我们发现有部分结节可进行性增大，而且有1例患者部分结节相互融合逐步转为条带型，有2例干性胸膜转移的患者在病情发展中出现恶性胸腔积液。Kim等^[5]^也指出这一点，并且研究显示胸膜转移患者从干性胸膜转移到出现胸腔积液的时间间隔从1个月-59个月不等（中位间隔时间19个月）。因此，我们推测结节型的胸膜转移可能是条带型转移的早期阶段。而干性胸膜转移可能是胸膜转移演变转化过程中的早期阶段^[5]^，而这需要更多病例和更长时间的随访进一步证实。

文献^[5]^报道，非小细胞肺癌患者有胸膜转移或伴有对侧肺恶性肺结节的5年生存期分别为2%和3%；中位生存期分别为8个月和10个月，而干性胸膜转移的中位生存期明显长于湿性胸膜转移，分别为38个月和13个月。因此，在患者初诊时检出干性胸膜转移就尤为重要，有助于临床医生采取较为积极的治疗手段和选择合适的治疗方案。

资料^[1]^显示，高达50%的干性胸膜转移患者实施了无效的手术治疗。MSCT具有较高的空间分辨率，尤其是薄层重建及多平重组图像，对干性胸膜转移的诊断有较高的准确性。而干性胸膜转移的检出可避免不必要的手术治疗。因此当非小细胞肺癌患者的CT影像学表现发现胸膜结节（尤其是多发叶间胸膜结节）或（和）胸膜增厚时，影像医生要警惕干性胸膜转移的可能，以免造成漏诊。
